# A reliability and validity study for Scolioscan: a radiation-free scoliosis assessment system using 3D ultrasound imaging

**DOI:** 10.1186/s13013-016-0074-y

**Published:** 2016-05-31

**Authors:** Yong-Ping Zheng, Timothy Tin-Yan Lee, Kelly Ka-Lee Lai, Benjamin Hon-Kei Yip, Guang-Quan Zhou, Wei-Wei Jiang, James Chung-Wai Cheung, Man-Sang Wong, Bobby King-Wah Ng, Jack Chun-Yiu Cheng, Tsz-Ping Lam

**Affiliations:** Interdisciplinary Division of Biomedical Engineering, The Hong Kong Polytechnic University, Hong Kong, People’s Republic of China; School of Public Health and Primary Care, The Chinese University of Hong Kong, Hong Kong, People’s Republic of China; Department of Orthopaedics and Traumatology, The Chinese University of Hong Kong, Hong Kong, People’s Republic of China

**Keywords:** Scoliosis, AIS, Cobb angle, 3D ultrasound, Volume projection imaging, Scolioscan

## Abstract

**Background:**

Radiographic evaluation for patients with scoliosis using Cobb method is the current gold standard, but radiography has radiation hazards. Several groups have recently demonstrated the feasibility of using 3D ultrasound for the evaluation of scoliosis. Ultrasound imaging is radiation-free, comparatively more accessible, and inexpensive. However, a reliable and valid 3D ultrasound system ready for clinical scoliosis assessment has not yet been reported. Scolioscan is a newly developed system targeted for scoliosis assessment in clinics by using coronal images of spine generated by a 3D ultrasound volume projection imaging method. The aim of this study is to test the reliability of spine deformity measurement of Scolioscan and its validity compared to the gold standard Cobb angle measurements from radiography in adolescent idiopathic scoliosis (AIS) patients.

**Methods:**

Prospective study divided into two stages: 1) Investigation of intra- and inter- reliability between two operators for acquiring images using Scolioscan and among three raters for measuring spinal curves from those images; 2) Correlation between the Cobb angle obtained from radiography by a medical doctor and the spine curve angle obtained using Scolioscan (Scolioscan angle). The raters for ultrasound images and the doctors for evaluating radiographic images were mutually blinded. The two stages of tests involved 20 (80 % females, total of 26 angles, age of 16.4 ± 2.7 years, and Cobb angle of 27.6 ± 11.8°) and 49 (69 % female, 73 angles, 15.8 ± 2.7 years and 24.8 ± 9.7°) AIS patients, respectively. Intra-class correlation coefficients (ICC) and Bland-Altman plots and root-mean-square differences (RMS) were employed to determine correlations, which interpreted based on defined criteria.

**Results:**

We demonstrated a very good intra-rater and intra-operator reliability for Scolioscan angle measurement with ICC larger than 0.94 and 0.88, respectively. Very good inter-rater and inter-operator reliability was also demonstrated, with both ICC larger than 0.87. For the thoracic deformity measurement, the RMS were 2.5 and 3.3° in the intra- and inter-operator tests, and 1.5 and 3.6° in the intra- and inter-rater tests, respectively. The RMS differences were 3.1, 3.1, 1.6, 3.7° in the intra- and inter-operator and intra- and inter-rater tests, respectively, for the lumbar angle measurement. Moderate to strong correlations (R^2^ > 0.72) were observed between the Scolioscan angles and Cobb angles for both the thoracic and lumbar regions. It was noted that the Scolioscan angle slightly underestimated the spinal deformity in comparison with Cobb angle, and an overall regression equation y = 1.1797x (R^2^ = 0.76) could be used to translate the Scolioscan angle (x) to Cobb angle (y) for this group of patients. The RMS difference between Scolioscan angle and Cobb angle was 4.7 and 6.2°, with and without the correlation using the overall regression equation.

**Conclusions:**

We showed that Scolioscan is reliable for measuring coronal deformity for patients with AIS and appears promising in screening large numbers of patients, for progress monitoring, and evaluation of treatment outcomes. Due to it being radiation-free and relatively low-cost, Scolioscan has potential to be widely implemented and may contribute to reducing radiation dose during serial monitoring.

## Background

Scoliosis is a spinal deformity in the coronal plane associated with vertebrae rotation in the transverse plane and abnormal curvature in the sagittal plane [1, 2]. Adolescent idiopathic scoliosis (AIS) is the most prevalent form of scoliosis affecting 3–4 % of kids in Hong Kong [3] and about 5 % in China according to a recent study [4], which is a comparable prevalence to other countries [5]. AIS is often diagnosed during the pubertal growth spurt between 10 and 14 years of age [6, 7]. Young patients with AIS are generally skeletally immature and at risk for curve progression, thereby requiring regular monitoring of curve progression [8]. Quantitative assessment of curve severity is also important to plan surgery and for monitoring prognostic and therapeutic outcomes [5, 9].

Cobb angle measurement [10] in the frontal plane derived from standing postero-anterior radiographs is the current gold standard for scoliosis evaluation and to inform decision making for treatment. However, taking radiographs has its own risks and drawbacks. Radiation over repeated exposure to radiographs may increase the risk of breast cancer in girls with scoliosis [11–13]. In addition, radiographic diagnostics in childhood has been shown to contribute significantly to leukemia and prostate cancer [14]. A recent study reported that the management decisions, which are made mainly based on Cobb angle, for AIS significantly affect patient radiation exposure, and it was therefore suggested that research for new imaging modalities with limited ionizing radiation should be undertaken [15]. Efforts have been made to reduce the radiation dose for scoliosis evaluation by using scanning radiography imaging [16]. A new imaging system using this method, named EOS, has been developed, and recent studies demonstrated that it can significantly reduce the radiation exposure with similar quality of images in comparison with conventional standing radiographs [17, 18]. The EOS system is relatively expensive for both the device and its operation and its installation still requires a large space and radiation shielding, thus its accessibility will not be high in the foreseeable future. In addition, it is difficult to make a EOS machine mobile or portable for screening high numbers of scoliosis patients.

Alternatively, different surface topographic methods have been used to estimate spine curvature using stereo cameras or finger palpation of spinous processes to achieve radiation-free assessment of scoliosis, however, it has been demonstrated that these methods are not accurate enough [19, 20]. A recent multicenter study showed that a newly developed surface topography system had a good reproducibility, but still poor correlations with Cobb angle, with R^2^ value of 0.5 and 0.25 for thoracic and lumbar scoliosis, respectively [21]. This category of technique suffers from the lack of internal anatomical information of the spine, thus its accuracy is inherently limited.

On the other hand, the feasibility of using various bony landmarks in B-mode images to evaluate spine deformity has been demonstrated previously [22]. Recently, freehand 3D ultrasound, combining conventional B-mode ultrasound with position sensors, has been advanced to overcome the limitations of 2D viewing and measuring of 3D musculoskeletal anatomy [23, 24], and a number of such systems have been reported for scoliosis assessment [25–29]. Different methods have been proposed to estimate spinal deformity using the 3D ultrasound data. In one method, spine curvature was estimated through manually locating the transverse processes in some ultrasound images with 3D spatial information. These ultrasound images were manually selected from a pile of recorded 2D raw B-mode images [30, 31] or captured in real-time while locating the target from observations [29]. This method is relatively time-consuming as each required body landmark has to be manually identified in B-mode images, but it can form a virtual 3D model of the whole or a part of spine using the detected landmarks. The second method is to measure the spine curvature based on the 3D volume ultrasound data, with different visualization methods for the spine anatomy, such as maximum intensity projection [28] and volume projection imaging (VPI) [32]. Koo et al. [33] compared different methods for measuring spinal curvature of spine phantoms using data collected with 3D ultrasound imaging.

The feasibility and potential of using the 3D ultrasound imaging methods have been clearly demonstrated in recent studies for the measurement of scoliotic deformity in vivo [31, 32, 34, 35] as well as for the improvement of brace fitting for scoliosis patients [36, 37]. Being a radiation-free and cost-effective imaging modality, ultrasound imaging has potential to be widely used for scoliosis assessment; its popularity will be enhanced with improved portability of the ultrasound scanner. However, all reported 3D ultrasound imaging systems have been experimental prototypes and not optimized for large scale clinical application. In addition, 3D ultrasound imaging for scoliosis evaluation involves steps of manual scanning and angle measurement, and reliability of each step has not yet been systematically evaluated. Therefore, many investigations are still required before 3D ultrasound imaging can become a clinical tool to benefit scoliosis patients.

The aim of this study was to investigate the reliability of a newly developed 3D ultrasound imaging system for scoliosis assessment, named as Scolioscan. Scolioscan uses volume projection imaging method [32] to form coronal view images of spine structure for the measurement of spinal curvature in the coronal plane. It is a system developed for clinical applications with designs to stabilize patient posture during scanning, but the reliability of using Scolioscan for the assessment of scoliosis patients has not been reported. The intra- and inter-operator reliability of scanning as well as intra- and inter-rater reliability of angle measurement of using Scoliosis were systematically tested in this study. The correlation between the angle measured using Scolioscan and the Cobb angle measured using conventional plain radiography was also investigated to demonstrate its validity. It is believed that the results of this study will provide a good reference for further research and clinical applications of using 3D ultrasound imaging for scoliosis.

## Methods

### Scolioscan system

**Fig. 1:**
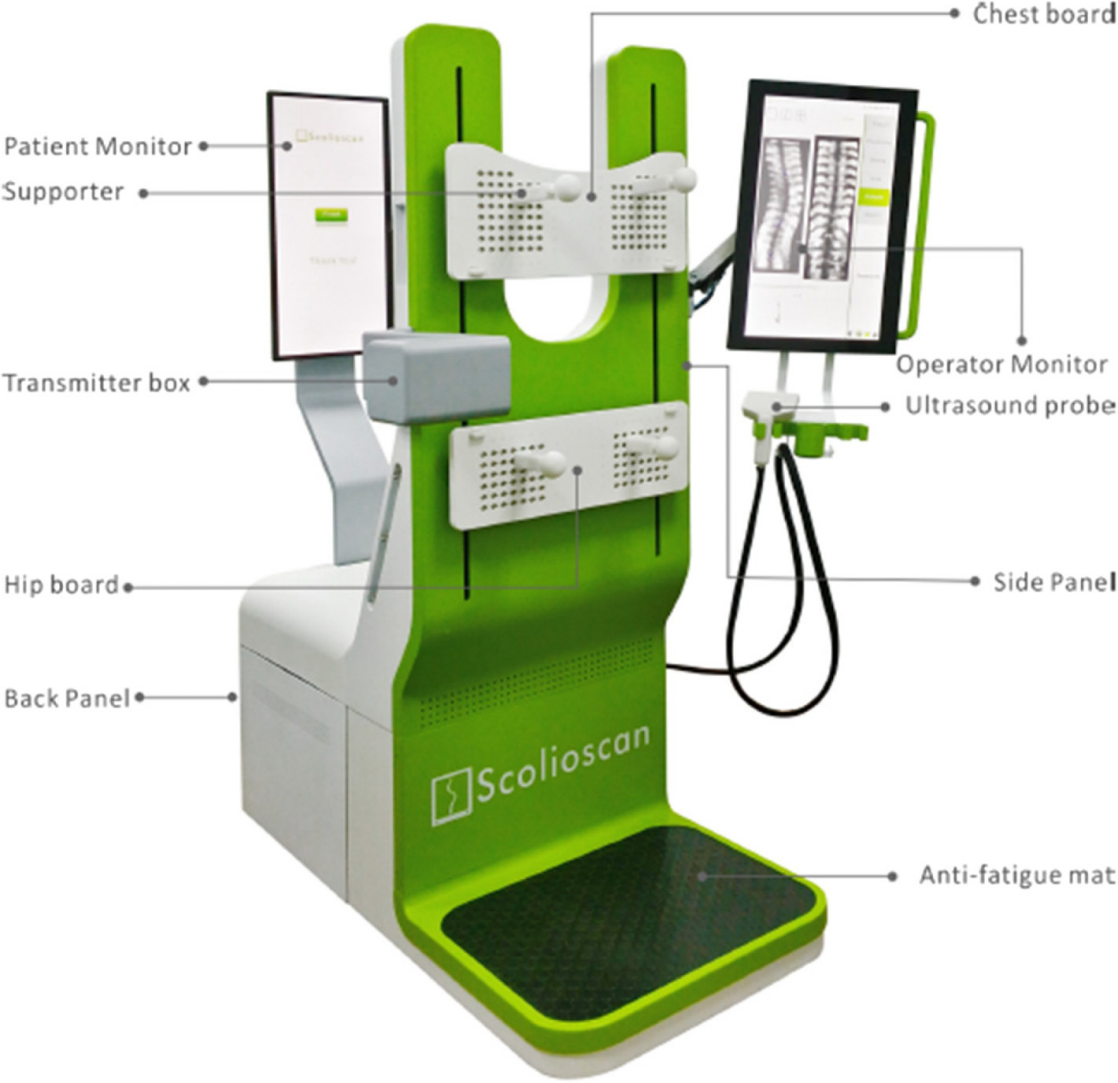
The Scolioscan system with its components labeled. The ultrasound scanner, computer and spatial sensor control box are installed inside the device

**Fig. 2:**
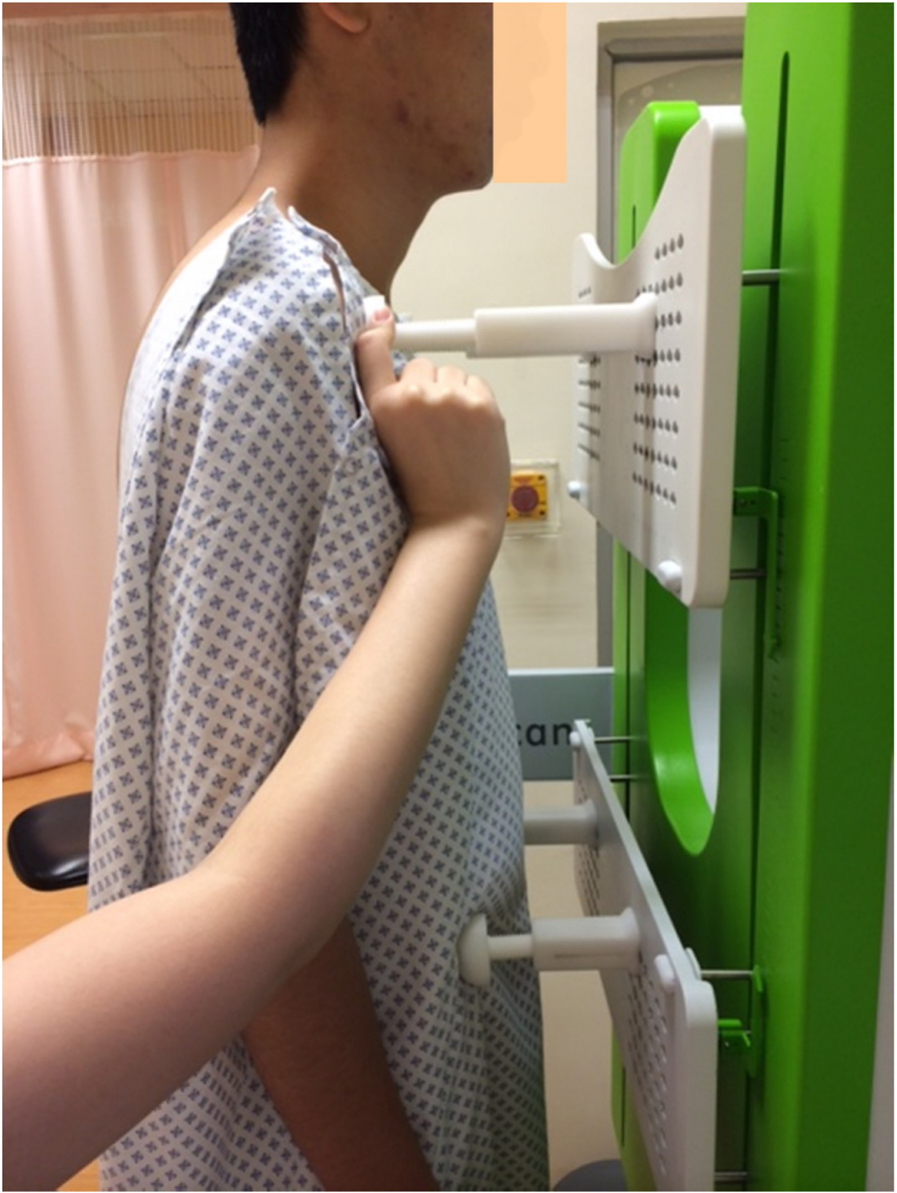
A subject being supported by the four supporters, with their locations being adjusted on the chest and hip boards and their lengths adjusted according to the need of each subject

**Fig. 3:**
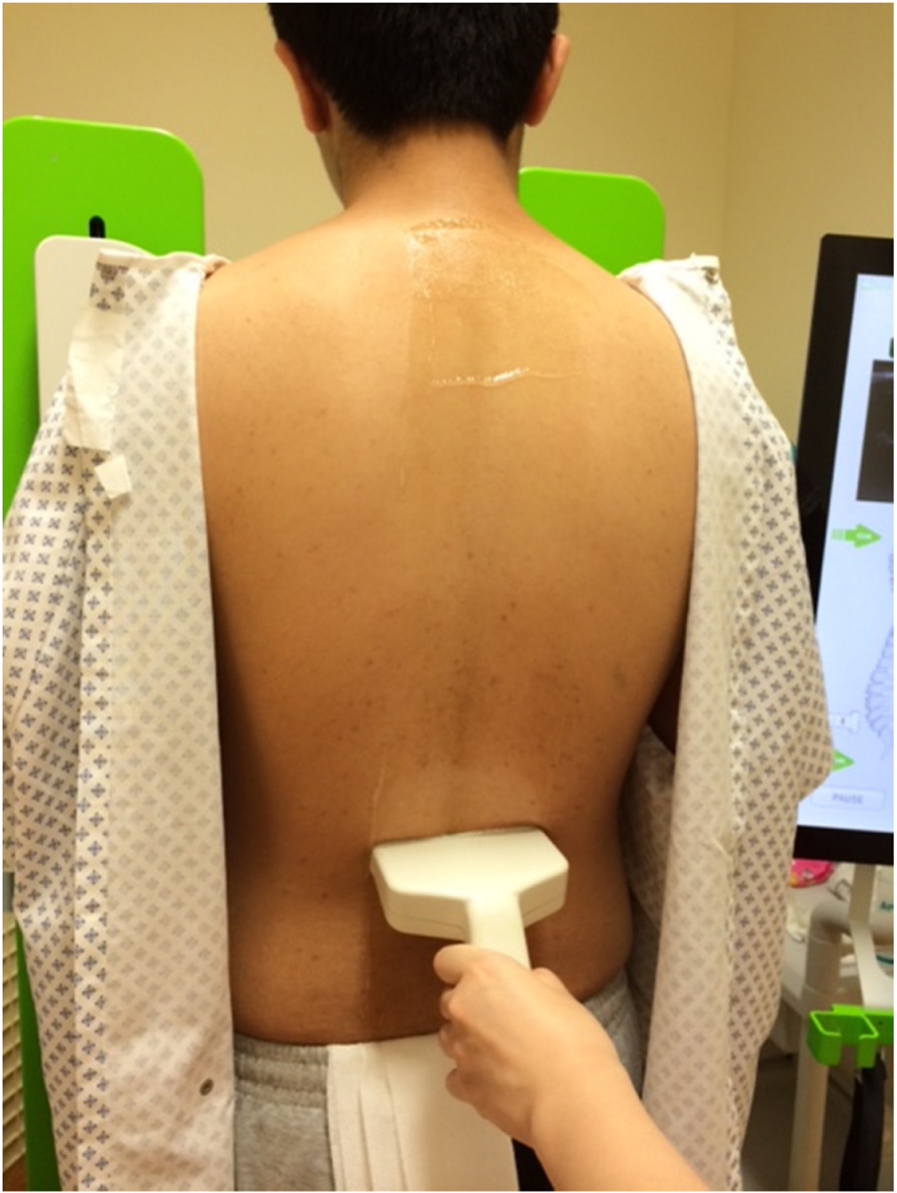
A subject being scanned by the Scolioscan probe, with supports provided by the four supporters installed on the chest and hip boards during scanning. Ultrasound gel is applied along the screening region

The Scolioscan system (Model SCN801, Telefield Medical Imaging Ltd, Hong Kong) was developed based on the 3D ultrasound imaging method reported earlier [25, 30–32], but with industrial and ergonomic designs of the hardware and software interfaces. As shown in Fig. 1, the system includes a rigid frame with two movable supporting boards and four supporters to support patients to maintain a stable posture during a test (Fig. 2). The chest and hip boards can be moved up and down to fit patients with different heights, and the four supporters with their length adjustable can be fixed on the boards by inserting to the fixation holes and locked by rotating the supporter by 90°. The locations of boards in vertical direction, the positions of supporters along vertical and horizontal directions, as well as the lengths of supporters can be recorded, and the information can be used in follow-up assessments for the same patient. The 3D ultrasound imaging of the spine is achieved through freehand scanning of the ultrasound probe (a custom-designed linear probe with frequency of 4–10 MHz and width of 10 cm), inside which an electromagnetic spatial sensor is installed to detect the position and orientation of the probe. The electromagnetic transmitter is located inside the transmitter box as indicated in Fig. 1. Figure 3 shows a subject being scanned, and the probe is moved from bottom to top of the back to cover the whole spine. The Scolioscan system has two LCD screens, with one touch screen in the front being used by the operator for inputting patient information, setting parameters for scanning, controlling image collection, data saving and retrieving, conducting VPI image formation, performing measurement, and generating reports. The other screen on the back is to provide information for patients, including a green eye-spot with location set according to the height of patient to facilitate him/her to keep a stable head and neck posture during scanning. This screen also shows additional information including different steps of evaluation procedures, so as to keep the patient informed about the process, and thus more cooperative.

**Fig. 4:**
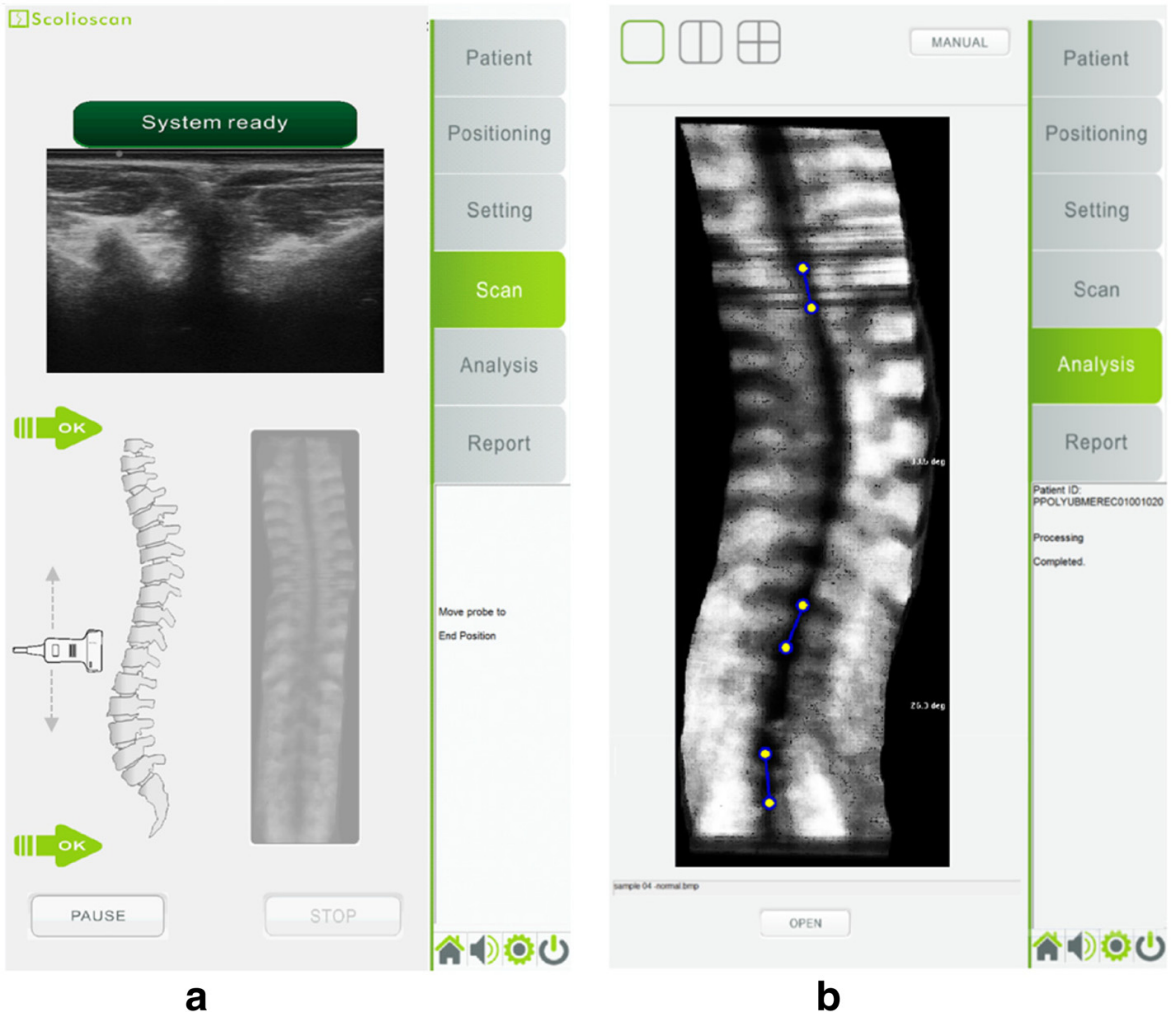
Typical software interfaces for (**a**) scanning and (**b**) VPI image analysis and angle measurement of Scolioscan. As shown in the tabs at the right side of the interface, other steps of the assessment procedure include: registration of patient information, adjustment of supporters, setting for ultrasound scanner, and reporting

Figure 4 shows the typical software interfaces for (a) scanning and (b) VPI image analysis and angle measurement of Scolioscan during measurement. Other steps of the assessment procedure include: registration of patient information, adjustment of supporters, setting for ultrasound scanner, and reporting. Before conducting a scan, the range of scanning is first determined by putting the ultrasound probe at the bottom side to record the lower boundary and then at the top side to record the upper boundary. During scanning, a moving probe is shown (real-time) in the interface to indicate the location of the ultrasound probe in relation to the upper and lower boundaries to guide scanning.

**Fig. 5:**
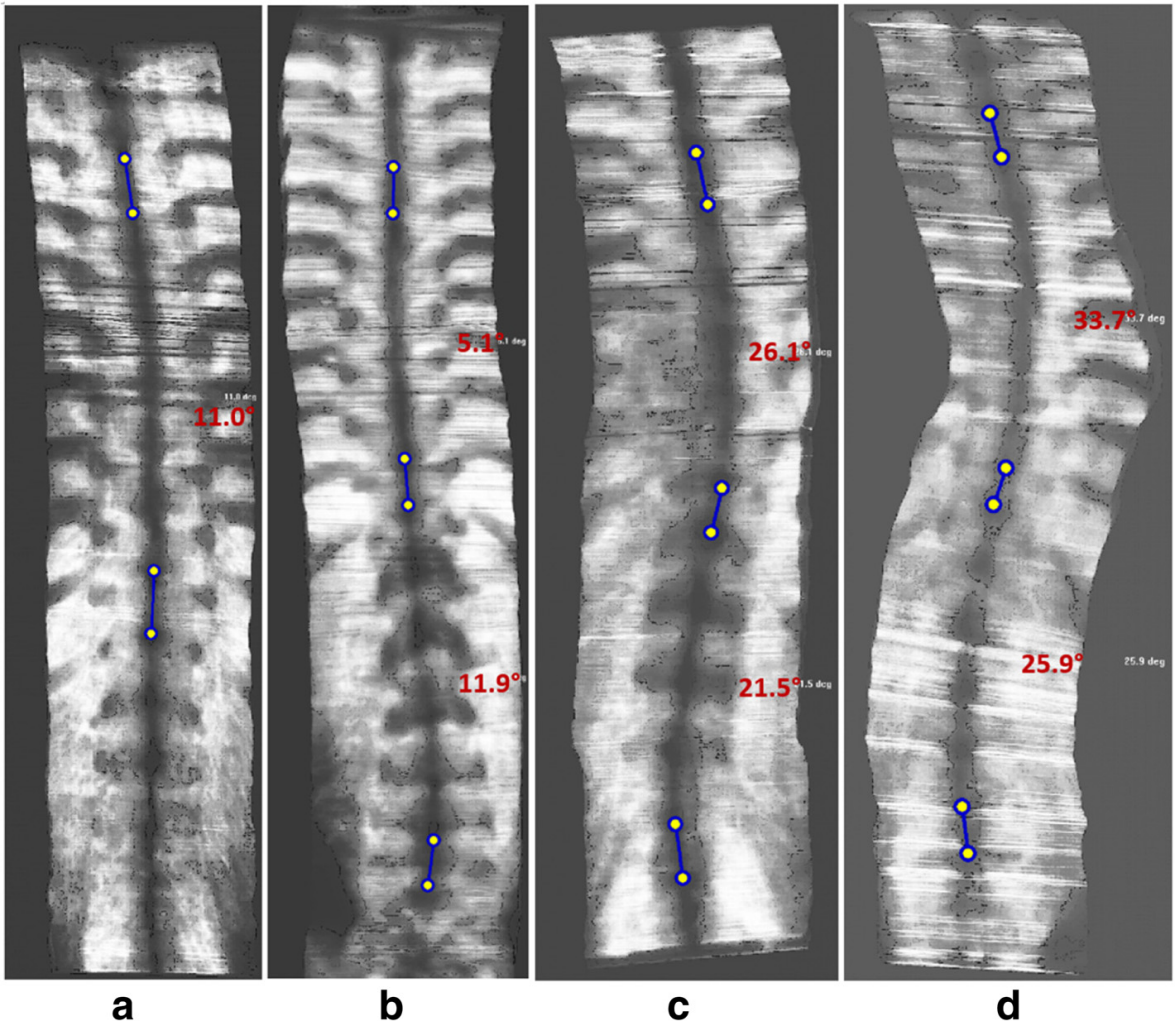
Four typical volume projection images obtained by Scolioscan showing the coronal plane of spine with different levels of deformity, with the two lines manually drawn to measure the curvature of the thoracic region (**a**), and the three lines to form two pairs to measure the curvatures of spine in the thoracic and lumbar regions, respectively (**b**, **c**, **d**)

After scanning, the collected B-mode image data together with the corresponding position and orientation information recorded are used for 3D image reconstruction, and volume project imaging is used to form coronal view images of the spine for further analysis [32]. The key idea of VPI method is to obtain an averaged intensity of all voxels of the volumetric image within a selected depth of approximately 10 mm along the antero-posterior direction to form an image in the coronal plane. In addition, a non-planar re-slicing technique is used to enhance the spinal profile in the coronal image by using the skin surface as a reference for selecting the required voxels. Figure 5 shows four typical VPI images obtained from patients with different severity of scoliosis. Thereafter, the curve found approximately near the mid-line of the volume projection image, which represents the location of spinous processes, is used to measure the spinal deformity angles. At least two greatest turning portions of a scoliotic curve could be identified as the most tilted vertebrae for angle measurement. Two short lines were then manually drawn from the middle of the curve on the coronal image for denoting the local turning of curve. The angle of the spinal curvature was automatically derived according to the orientations of the two lines drawn, and it was named as Scolioscan angle for this paper (Fig. 5). For example, if the spine has an S-shape three lines would be drawn on the VPI image to measure thoracic and lumbar Scolioscan angles.

### Subjects

Patients diagnosed with scoliosis and scanned by radiographs were invited for this study. All AIS patients were recruited consecutively in the Department of Orthopaedics and Traumatology of The Chinese University of Hong Kong. The study got human subject ethical approvals from both The Hong Kong Polytechnic University (No. 20070321001) and The Chinese University of Hong Kong (No. 2009.622). Informed consent was obtained from all patients (or their parents for those under 18 years of age). The patients received conventional standing plain radiographs within three months before the Scolioscan assessment, and was used for Cobb angle measurement. Patients with metallic implants and BMI higher than 25.0 kg/m^2^ were excluded, as a high BMI may lead to poor image quality in the lumbar region using the current probe and the metallic implants may potentially affect the accuracy of ultrasound probe spatial sensing, which uses electromagnetic fields. In addition, patients with Cobb angle larger than 50° were also excluded. Others were further excluded due to following reasons: 1) Patient refused continuation during scanning; 2) Appearance of darkened areas in some of VPI images due to winged scapula, which affected a smooth scanning; 3) Allergy to ultrasound gel; and 4) Patient who wore a bracelet during the final radiograph. One patient felt dizzy during the scanning and subsequent scans were canceled, and five patients had severe winged scapula resulting in poor images so their data were excluded (out of total 55 subjects tested). These data were excluded from analysis.

Twenty patients were included for the first stage of intra-/inter-operator and intra-/inter-raters reliabilities study (with four male and 16 female subjects; age range of 12–22 years of age, mean of 16.4 ± 2.7 years; BMI range of 16.0–22.3 kg/m^2^, and mean of 18.6 ± 1.6 kg/m^2^). For the second study stage examining correlation between Scolioscan and X-ray measurements, additional patients were recruited, making the total patient number of 49 (with 15 male and 34 female; age range of 11 to 23 years of age, mean 15.8 ± 2.7 years; BMI range 15.1–23.9 kg/m^2^, mean 18.4 ± 1.7 kg/m^2^).

### Testing protocol

The patient was requested to undress upper garments and shoes before the scanning session and was provided a back-opening dressing gown for ease of scanning. All metallic objects, electronics goods, magnets, and other possible ferromagnetic materials were removed. The patient was asked to stand on the Scolioscan platform for supporter adjustment. The chest and hip boards were repositioned at his/her reasonable height (Figs. 1, 2). Two supporters on the chest board were relocated to align with clavicle anterior concavities; whereas two supporters on the hip board were relocated to align with bilateral anterior superior iliac spines, the length of supporter’s shafts on both boards was adjusted until they came in contact with the patient. The patient was instructed to maintain their natural standing posture after the adjustment of supporters, and to keep their eye level horizontal at the level of the eye-spot shown on the patient screen and to focus on the spot throughout the scanning process.

The operator applied warmed aqueous ultrasound gel to the patient’s back to fill the spinal furrow and cover the extent of where the probe would sweep. Now the patient was ready to be scanned by the probe, and then ultrasound scanner and the spatial sensing system were activated. The TGC and brightness of B-mode ultrasound images could be adjusted according to the tissue condition of each patient, and like a conventional B-mode ultrasound scanner, other adjustments including scanning depth, focus, frequency, etc. were possible. Parameters were fixed for the tests of all subjects, with the frequency set at 7.5 MHz and depth at 7.1 cm. To increase the B-mode imaging frame rate, a single focus was used and set at depth of 3.5 cm. Pre-scanning was performed from L5 to T1 to check the image quality, and corresponding adjustment of TGC and brightness for B-mode image was conducted to achieve an overall good image quality for the scanning region.

After the setting adjustment, the probe was located at level L5 and T1 spinous processes to record the lower and upper boundaries of scanning range, respectively. The operator used their finger to touch the two green arrows in the “Scan” interface shown on the operator screen to record the probe location information (Fig. 4a). After the scanning range was set, the operator re-located the probe at the location slightly lower than L5 spinous process and initiated the scanning and steered the probe to scan upwards from L5 to T1 spinous process. During scanning, an additional arrow would be shown in the interface to indicate the location of the probe in relation to the upper and lower boundaries of scanning range. The data collection was automatically stopped when the probe passed through the upper boundary. The recorded data was then saved with file name containing information of patient code and scanning time, unless the operator decided to discard the scanning result. Once the data were saved, the process of VPI image formation was automatically initiated and user interface was changed from “Scan” to “Analysis” (Fig. 4). The procedure of using the probe to scan over the spine region takes approximately 30 s, and VPI image formation less than two minutes for the Scolioscan system used in this study. The total time for assessing one patient was approximately 10 min, including time required for inputting patient information in Scolioscan, supporter adjustment to fit patient, identifying landmarks, applying ultrasound gel, scanning, image reconstruction, measurement on image, and reporting.

### Study design

This prospective study was divided into two stages. At the first stage, the intra- and inter-operator reliability for scanning as well as the intra- and inter-rater reliability for measurement using Scolioscan were investigated. At the second stage, the correlation between Cobb angle obtained from radiographs and spinal curve angle obtained using Scolioscan was investigated.

**Fig. 6:**
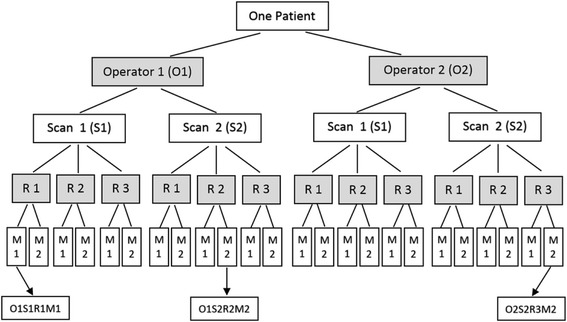
Schematic diagram showing the experimental design for evaluating the intra- and inter-reliability for the two operators for scanning and three raters for angle measurement on images. “R” represents “Rater”, and “M” represents “Measurement”. For each patient, a total of 24 sets of measurement result were obtained. Each result is represented as “O*S*R*M*”, with “O”, “S”, “R”, and “M” represent “Operator”, “Scan”, Rater”, and “Measurement”, respectively. For example, “O1S2R2M2” represents the result obtained from the second measurement of Rater 2 for the image obtained during second scanning of the Operator 1

For the spine scanning session using the Scolioscan system at the first stage, two operators (KKL and SY) were involved for the scanning and each AIS patient received scanning twice by each of the operators. For each scanning of each operator, a VPI image was formed, which was then viewed by three raters (TTY, KKL, and SY) to conduct spine curvature measurement independently using the manual measurement tool provided by the Scolioscan system in the interface of “Analysis” (Fig. 4). Each VPI image was measured twice by each rater, but not at the same time. Two of the raters were also the operators, and both the operators and the raters were blinded from each other for the scanning and measurement. Before the scanning/measurement started, there was a trial tutorial for all the operators/raters to have a common understanding of the scanning and measurement procedure. The entire procedure for the first stage is summarized in Fig. 6. For each patient, a total of 24 sets of results were generated.

For the investigation of correlation between the Scolioscan angles and the radiographic Cobb angles at the second stage, the scanning on patients using Scolioscan was conducted by a single operator (Operator 1 also Rater 2 at the first stage; KKL) and the measurement of spinal curvature on the obtained VPI image was conducted by the same person. Only a single measurement was conducted for each image, which is the same as Cobb angle measurement on X-ray images. The Cobb angles were measured by a doctor in the orthopedics department (TP) who has over 10 years of experiences in reading radiographs of patients with scoliosis. The CV% of his Cobb angle measurement was 6.4 %. Angles were categorized as the thoracic Cobb angle if the apex vertebra located within T1-T12 region in X-ray images or as the lumbar Cobb angle if the apex vertebra located within L1-L5 region, and such categorization was also used in the angle measurement of the Scolioscan measurement.

### Statistical analysis

At the first stage of the study, the ICC (two-way random and consistency) was used to analyze the reliability between the two sessions of the same rater and operator [38]. All the tests and corresponding data sets used are summarized in Table 1. For the intra-rater reliability, two measurements acquired from the first scan by each operator were compared individually for each rater. For the intra-operator reliability, the first measurement of the first scan was compared with that of the second scan for each of the two operators. ICC with two-way random and absolute agreement was used to analyze the reliability between the two sessions of the different raters and operators [38]. For the inter-operator reliability, the first measurement results obtained by each of the three raters from the first scan of the two operators were compared. While for the inter-rater reliability, the first measurement results from the first scan of each of the two operators conducted by the three raters were compared. The Currier criteria for evaluating ICC values were adopted: very reliable (0.80–1.0), moderately reliable (0.60–0.79), and questioned reliable (≤ 0.60) [39]. According to the result of a pilot study, we expected a high reliability (ICC = 0.9) of the measurement using Scolioscan. We further assumed that achieving a moderately reliable result (ICC = 0.7) was meaningful in this study. Thus we were able to calculate the minimum subject numbers required to be 18 for two operators/raters and 12 for three operator/raters, assuming a power of 80 % [40]. Accordingly, 20 subjects were recruited for the reliability test in this study. RMS difference was calculated for every pair of intra- and inter-operator as well as intra- and inter-rater reliability tests to provide additional information about test repeatability.

**Table 1:** Data sets used in different tests for reliability, with “O”, “S”, “R”, and “M” represent “Operator”, “Scan”, Rater”, and “Measurement”, respectively (Fig. 6). Each set of result is represented as “O*S*R*M*”. For example, “O1S2R2M2” represents the result obtained from the second measurement of Rater 2 for the image obtained during second scanning of the Operator 1

Reliability Test	Exam	Result Table	Remarks
Intra-rater	O*S1R*M1, O*S1R*M2	Table 1	Tests between the two measurements by each rater (R*) using the first scan of each operator (O*), and for each region (thoracic and lumbar)
Intra-operator	O*S1R*M1, O*S2R*M1	Table 2	Tests between the two scans obtained by each operator (O*), using the first measurements of each other three raters (R*), and for each region
Inter-rater	O*S1R1M1, O*S1R2M1, O*S1R3M1	Table 3	Tests among the first measurements of the three raters using the first scan of each operator (O*), and for each region.
Inter-operator	O1S1R*M1, O2S1R*M1	Table 4	Tests between the two operators using the first scan, with the first measurement of each of three raters, and for each region

At the second stage, the Scolioscan angles and radiographic Cobb angles were compared using linear correlation for thoracic curves alone, lumbar curves alone, and combined results. Linear regression equations with and without intersections were analyzed, with correlation coefficient 0.25 to 0.50 indicating poor correlation, 0.50–0.75 indicating moderate to good correlation, and 0.75–1.00 indicating very good to excellent correlation [41]. According to the results of other radiation-free assessment method for scoliosis, achieving a moderate to good correlation (correlation coefficient = 0.55) between the Scolioscan angle and radiographic Cobb angle would be meaningful for this study. Thus, the sample size required was 24 to achieve a power of 80 % [42]. Considering that some patients may only have either thoracic or lumbar curve, the patient number was determined to be 48. In this study, 49 patients were finally recruited for the correlation study. Bland-Altman method was used to test the agreement between the Cobb angle and the Scolioscan angle. The RMS difference between Scolioscan and Cobb angles was also calculated to show the agreement between the results of the two methods, for the cases with and without correction using the regression equation.

## Results

The results demonstrated that the coronal image of spine could be successfully obtained for all the patients tested in this study. The mean Cobb angles of the patients involved in the reliability tests were 30.0 ± 12.3° (mean ± SD, range 11 to 48°, 12 angles) and 24.8 ± 11.0° (seven to 46°, 14 angles) for the thoracic and lumbar regions, respectively, and the overall mean was 27.6 ± 11.8° (seven to 48°, 26 angles). The intra-rater reliability of Scolioscan angle measurement was very good with ICC ranging from 0.94 to 0.99 (0.97 ± 0.02), for each of the three raters conducting measurement for thoracic and lumbar region using the scan of the two operators individually (Table 2). Table 3 shows a very good intra-operator reliability with ICC ranging from 0.88 to 0.97 (0.94 ± 0.03), for each of the two operators with the angle measurement conducted by the three rater individually. The results demonstrated that Scolioscan provided very good reliability for the scanning by the same operator and the angle measurement by the same rater. The RMS differences between the angles obtained from the two scans of the same operator were 2.5 and 3.1° for the thoracic and lumbar regions, respectively (counting the results of the two operators and three raters for a single measurement of each image). The RMS differences of the intra-rater tests were 1.5 and 1.6° for the thoracic and lumbar regions, respectively.

**Table 2:** Intra-rater reliability of the three raters individually for the curve measurement performed using the images scanned by the two operators in the thoracic and lumbar regions using Scolioscan

		Thoracic		Lumbar	
Operator		Operator 1	Operator 2	Operator 1	Operator 2
ICC^a^	Rater 1	0.99	0.99	0.98	0.94
	Rater 2	0.98	0.94	0.97	0.96
	Rater 3	0.99	0.98	0.96	0.97

^a^ *ICC* intraclass correlation coefficient

**Table 3:** Intra-operator reliability of the two operators individually for the curve measurement performed by the three raters in the thoracic and lumbar regions using Scolioscan

		Thoracic			Lumbar		
Rater		Rater 1	Rater 2	Rater 3	Rater 1	Rater 2	Rater 3
ICC^a^	Operator 1	0.97	0.96	0.96	0.90	0.92	0.97
	Operator 2	0.97	0.92	0.95	0.91	0.88	0.96

^a^ *ICC* intraclass correlation coefficient

The results also showed very good inter-rater reliability for angle measurement and inter-operator reliability for scanning using Scolioscan, with ICC values ranging from 0.88 to 0.93 (0.90 ± 0.02) and 0.87 to 0.94 (0.92 ± 0.03), respectively (Tables 4 and 5). The RMS differences between the angles obtained from the scans of the two operators were 3.3 and 3.1° for the thoracic and lumbar regions, respectively (counting the results of three raters for a single measurement of one image). The RMS differences of the inter-rater tests were 3.6 and 3.7° for the thoracic and lumbar regions, respectively. The reliability results demonstrated that both scanning and angle measurement on VPI images for scoliosis patients were repeatable using the Scolioscan system, with the RMS difference between any two measurements or two scans smaller than 3.7°.

**Table 4:** Inter-rater reliability among the three raters for the curve measurement performed using the images scanned by the two operators in the thoracic and lumbar regions using Scolioscan

	Thoracic		Lumbar	
Operator	Operator 1	Operator 2	Operator 1	Operator 2
ICC^a^	0.93	0.89	0.89	0.88

^a^ *ICC* intraclass correlation coefficient

**Table 5:** Inter-operator reliability between the two operators for the curve measurement performed by the three raters in the thoracic and lumbar regions using Scolioscan

	Thoracic			Lumbar		
Rater	Rater 1	Rater 2	Rater 3	Rater 1	Rater 2	Rater 3
ICC^a^	0.94	0.87	0.95	0.93	0.88	0.94

^a^ *ICC* intraclass correlation coefficient

**Fig. 7:**
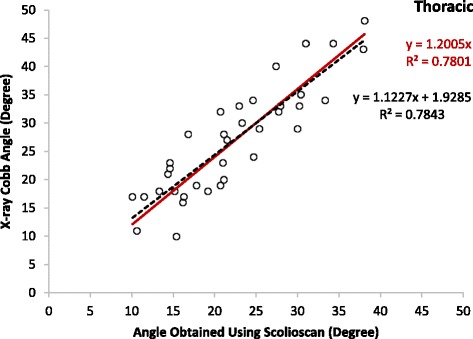
Correlation between the Cobb angles obtained using radiographs and the spinal angles measured using the coronal images generated by Scolioscan for the thoracic region

**Fig. 8:**
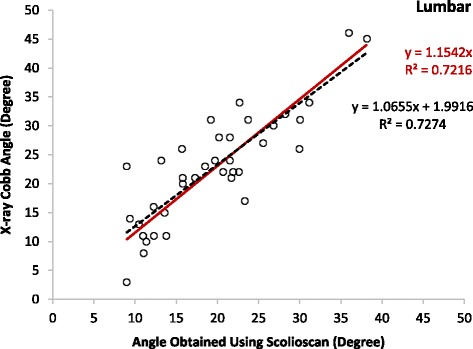
Correlation between the Cobb angles obtained using radiographs and the spinal angles measured using the coronal images generated by Scolioscan for the lumbar region

**Fig. 9:**
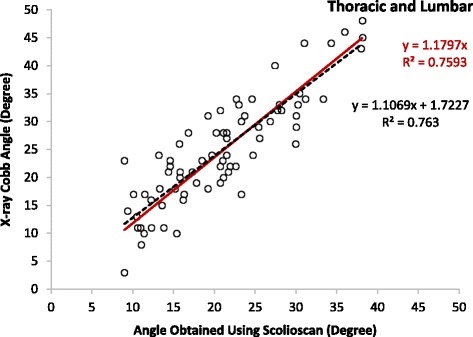
Correlation between the Cobb angles obtained using radiographs and the spinal angles measured using the coronal images generated by Scolioscan for both the thoracic and lumbar regions

**Fig. 10:**
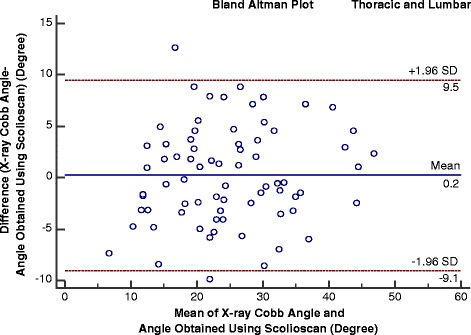
The Bland-Altman plot for the Cobb angles and the Scolioscan angle corrected using the linear regression equation for data combined with the thoracic and lumbar regions

The mean Cobb angles of the patients involved in the correlation tests were 26.9 ± 9.7° (10 to 48°, 36 angles) and 22.6 ± 9.5° (three to 46°, 37 angles) for the thoracic and lumbar regions, respectively, and the overall mean was 24.8 ± 9.7° (three to 48°, 73 angles). The results showed that there were moderate linear correlations between the Scolioscan angles and Cobb angles for the thoracic (y = 1.2005x, R^2^ = 0.78) and lumbar regions (y = 1.1542x, R^2^ = 0.72) and thoracic-lumbar data combined (y = 1.1797, R^2^ > 0.76) (Figs. 7, 8 and 9). It was noted that the Scolioscan angle slightly underestimated the spinal deformity in comparison with Cobb angle for both the thoracic and lumbar regions. When the linear regressions with intersection were used for the correlation, the intersection values were 1.93, 1.99, and 1.72°, for the thoracic region, lumbar region, and combined data, respectively. The small intersection values indicated that linear regressions without intersection could well represent the relationship between the Scolioscan angle and Cobb angle for the patients measured in this study. This could also be verified by the very small difference of the coefficients of determination R^2^ between the two kinds of linear regressions, as shown in Figs. 7, 8 and 9. In addition, it was found that the regression curves were very close between the angles of the thoracic and lumbar regions. Therefore, a single equation derived from the data combined with thoracic and lumbar regions was good enough to represent the relationship between the Scolioscan angle and Cobb angle regardless of the regions for the patients tested in this study (Fig. 9). The equation y = 1.1797x (R^2^ = 0.76) could be used to transfer the Scolioscan angle (x) to Cobb angle (y) for this group of AIS patients. The Bland-Altman method was used to test the agreement between the data of Cobb angle and those of the Scolioscan angle corrected by this equations, with results showing in Fig. 10. A very good agreement was demonstrated between the two types of angle, with a mean difference of 0.2°. The RMS differences between the Scolioscan angles and Cobb angles were 6.5, 5.9, 6.2°, respectively, for the thoracic, lumbar, and two regions combined cases. If a correction was conducted using the overall regression equation y = 1.1797x, the RMS difference became 4.5, 5.0, and 4.7°, respectively, for the three cases.

## Discussion

The results of our study showed very good reliability of Scolioscan for scanning conducted by the same and different operators as well as for the angle measurement performed by the same and different raters on the formed coronal spine images, with a mean ICC value of 0.94 ± 0.04 (ranging from 0.88 to 0.97) between the two operators and among the three raters. The high intra- and inter-rater reliability for the angle measurement showed in this study was consistent with that reported earlier in a feasibility study of VPI method [32]. The RMS difference was smaller than 3.7° between the angles obtained from different measurements or different scans of the same patient. The sophisticated supporting frame, boards, supporters, and eye guiding spot on the patient screen within Scolioscan’s design may have partially contributed to the very good intra- and inter-operator reliability for scanning (with re-positioning for each scan), with the RMS difference smaller than 3.3°. The two Scolioscan operators/raters had been using the system for several months before this study, and the additional rater had been trained for a few days for the measurement. They were all graduates from Biomedical Engineering programmes with knowledge of ultrasound imaging. It will be worthwhile to understand the learning curve of new operators with different backgrounds in future studies.

Once a patient is confirmed with scoliosis, they have traditionally had to be exposed to radiography many times for monitoring, treatment planning, and treatment outcome measurement [9]. With the use of radiation-free Scolioscan, many of the radiation exposures may be avoidable, such as those used for progression monitoring, which may reduce the risk of inducing cancers [11–15]. As a consequence of this radiation hazard, it is conventionally not possible to use frequent radiography for monitoring scoliotic angle progression, thus there is no reference yet about the optimal frequency of taking image for scoliosis patients using the radiation-free Scolioscan system. It may be worthwhile to conduct investigations along this direction, with the consideration of the angle progression rate, risk factors, and cost effectiveness for different categories of patients.

Moderate to strong linear correlations were demonstrated between the Scolioscan angles and X-ray Cobb angles for the thoracic and lumbar regions and thoracic-lumbar data combined with coefficients of determination R^2^ larger than 0.72. Similar results were reported earlier using different laboratory prototypes of 3D ultrasound imaging system for scoliosis assessment [26, 30, 31, 34, 36]. It was found that the Scolioscan angle slightly underestimated the spinal deformity in comparison with Cobb angle for both the thoracic and lumbar regions. For the patients tested in the present study, the relationship between the Scolioscan angle (x) to Cobb angle (y) could be expressed by the equation y = 1.1797x (R^2^ = 0.76). This finding is consistent with that reported previously using 3D ultrasound imaging for scoliotic angle measurement. The main reason for underestimation is that ultrasound images are taken posteriorly and provide anatomical features of vertebra posterior elements [32, 36, 43] rather than the vertebral bodies used in Cobb angle measurement from radiographs, as the processes are more identified than other spinal landmarks because of its sharp delineation in the ultrasound images. In this study, the profile formed by spinous processes in the VPI image was used for the deformity angle measurement. The RMS square difference between Scolioscan and Cobb angles obtained in this study (totally 73 angles) was 6.2°, and it became 4.7° when an adjustment for the Scolioscan angle was adopted using the obtained regression equation.

It has been well documented that the angle measured based on the profile of spinous processes in radiographs would underestimate the spinal deformity with reference to Cobb angle, showing that the spinous process angle was less angulated compared to Cobb angles [44]. It was reported that the magnitude of vertebral axial rotation correlated with the lateral deviation of vertebrae from the spinal axis [45, 46]. In fact, the spinous process deviations caused by vertebral rotation might result in the inaccuracy of interpretation on the vertebral body alignment on the radiographs of spine [47, 48]. A number of studies investigated how to transfer the spinous process angle to Cobb angle. An equation of y = 1.3367x + 1.3907 (R^2^ = 0.90) was proposed [44], with x representing the spinous angle and y the Cobb angle and both measured using radiographs. In the present study, the corresponding equations were y = 1.1069x + 1.7227 (R^2^ = 0.76) and y = 1.1797x (R^2^ = 0.76) for the regression with and without intersection (Fig. 9). In the earlier feasibility study about angle measurement using VPI images, the results from 3D ultrasound was closer to Cobb angles, where the involved patients had a much smaller mean Cobb angle of 10.7 ± 7.1° [32], in comparison with the mean Cobb angle of 22.6 ± 9.5° in the present study. Further studies for Scolioscan with larger patient numbers and a wider range of Cobb angle would be necessary to investigate whether different regression equations should be used for scoliosis subjects with different Cobb angles and different types of spinal deformity.

Future studies can also be followed up to understand whether considering the vertebral rotation and other spinal deformities can further improve the agreement [49]. AIS is a three-dimensional spine deformity problem in coronal and sagittal planes and vertebral rotation [1], and deformity parameters in different planes may be dependent on each other [50–52]. Therefore, it is necessary to quantify spinal curvatures in sagittal or vertebral rotation in addition to coronal deformity, which will be useful for planning surgery, predicting prognosis and monitoring curve progression [30, 53, 54]. A recent study showed that the correlation between the spinous angle and the Cobb angle measured on radiographs could be improved with the consideration of vertebral rotation [49]. However, standing radiograph as the current gold standard for scoliosis investigation is difficult to directly acquire vertebral rotation, since these radiographs do not demonstrate the exact magnitude of the 3-dimensional spinal deformity present in patients with scoliosis [55]. Using the information obtained from the coronal and sagittal radiographs with reduced dose, EOS system can reconstruct 3D view of spine [17, 18]. However, it may still take some time to make the system more popularly used because of its high cost, low accessibility, and radiation (though with dose reduced), and long time required for building 3D spine model. Furthermore, its 3D presentation of the spine achieved using two orthogonal projection images requires further research to validate for different cases. Scolioscan used in the present study provided VPI images of spine for spinal deformity measurement in the coronal plane. During the scanning, Scolioscan actually acquires volumetric images of spine. It has been demonstrated in earlier studies that it is feasible to extract bony landmarks from the volumetric image data set to form virtual 3D spine model for the assessment of scoliotic deformity [25, 30, 31]. Further studies are going on to integrate this function into the system so that the evaluation of scoliotic deformity in 3D can be achieved using Scolioscan, including the measurement of spinal axial rotation and the deformity in sagittal plane. In this study, patients with Cobb angle larger than 50° were excluded due to the concern of the effect from rotation. Perhaps if the spinal rotation can be measured, future studies can include patients with larger Cobb angles.

While the reliability of using the VPI images generated by Scolioscan for the scoliosis assessment has been clearly demonstrated in this study, there are a number of areas to improve so as to achieve a more user-friendly clinical tool. First, patients with AIS are often observed to have winged scapula, and the protruded scapula obstructed the probe from scanning upwards even when the patients were told to cross arm. Hence the quality of VPI image was affected, making it difficult for accurate measurement of angle. In this study, the patients with severe winged scapula that affected scanning were excluded, which counted for approximately 10 % of the patients. In future studies, ultrasound probes with different widths and shapes may be used to find optimal configurations for different situations. In addition, patients with BMI larger than 25.0 kg/m^2^ were excluded from this study, counting approximately 10 % of patients. The current Scolioscan system used an ultrasound probe with frequency of 4–10 MHz, and bony features in images of the lumbar region of subjects were affected by the thick tissue layer in high BMI patients due to its attenuation to the ultrasound signals. One potential solution is to use a probe with relatively lower ultrasound frequency for obese patients, with the trade-off of reduced image resolution. Further study is necessary to investigate the optimized ultrasound frequencies for patients with different BMI with the consideration of tissue penetration and image resolution simultaneously.

Second, the VPI images provided by Scolioscan show many more features than the profile of spinous processes used in the present study, but they have not been used for the analysis of spinal deformity. As shown in the images in Fig. 5, transverse processes and ribs can be observed in most of the VPI images. The feasibility of using transverse processes for the spinal deformity measurement has been demonstrated earlier based on VPI images [32]. Further studies would be worthwhile to follow up on how to utilize more features in VPI images to provide more parameters related to spinal deformity, including vertebral rotation. Since ultrasound images also recorded information of paraspinal muscle architecture, it will also be valuable to extract muscle related parameters for scoliosis assessment. In addition, the coronal images formed by the current Scolioscan only covered lumbar and thoracic regions, and not the whole spine structure. Therefore, the overall spinal alignment as well as the thoracic shift cannot be assessed yet. Further developments and studies are required to enable Scolioscan to provide more information of the whole spine structure, which will further widen its application.

Third, it was found that the VPI image formation took between one and two minutes dependent on the height of the patient. While this is acceptable, it would be helpful if the VPI image can be provided immediately after the scanning so that the image quality can be confirmed and the patient can be discharged immediately after scanning. Related developments are underway and it has been demonstrated that real-time image formation is feasible using the system.

Fourth, the discrepancy between Cobb’s angle and Scolioscan angle could arise from the different time and day used for conducting both images. The inclusion criteria used in this study was shorter than 3 months between the two, with most of them within two weeks of each other. There may be some changes of angle during the period, and thus room for improvement exists.

Fifth, while the manual measurement of angle using VPI images appears very repeatable as demonstrated by the three raters in the present study, it reamins a subjective method. Although the three raters were mutually blinded for the measurement, they were in the same research team. Thus there was some common understanding about how to draw the lines on VPI images among the three raters. This may not be the case when Scolioscan is used in different clinical units, and different users may have different methods for drawing lines to measure angles. This may make the results difficult to compare among different clinical or research groups. This issue is not unique for obtaining Scolioscan angles in VPI images and can likely be overcome with clear operation and measurement guidelines. Radiographic Cobb angle measurement has been facing the same challenge, but it will greatly facilitate the measurement of spinal deformity angle based on VPI images if an automatic method can be developed. Related development work is ongoing.

## Conclusions

In conclusion, this is the first study to report on the development and human application of Scolioscan in assessing its reliability and validity for scoliosis assessment. The measurement using Scolioscan was demonstrated to be very reliable and good to excellent correlation noted in comparison with the conventional radiographic Cobb’s method. Since Scolioscan is radiation-free and readily accessible, it has the potential to be used to screen large numbers of patients with AIS to monitor progress and outcome of treatment, and with prognostic implications. Further studies are required to demonstrate its clinical values with a larger number of scoliosis patients with different types of curvature and the feasibility of automatic Scolioscan angles measurement. It would also be necessary to investigate the potential of axial rotation and sagittal measurement using Scolioscan. While the current Scolioscan system is relatively large in dimension, it is believed that a portable or even palm-size Scolioscan system will be available in the near future. Scolioscan with its further development may greatly facilitate AIS screening, an important and valid step for managing scoliosis [3, 56], as well AIS prognosis and progression monitoring.
